# Supplementary material for: The conformational landscape of TRiC ring-opening and its underlying stepwise mechanism revealed by cryo-EM
Source: QRB Discov. 2024 Dec 16;6:e7. doi: 10.1017/qrd.2024.17 (PMC11894413; doi:10.1017/qrd.2024.17)
Supplement: Jin et al. supplementary material [file S2633289224000176sup001.zip › supplement.pdf]

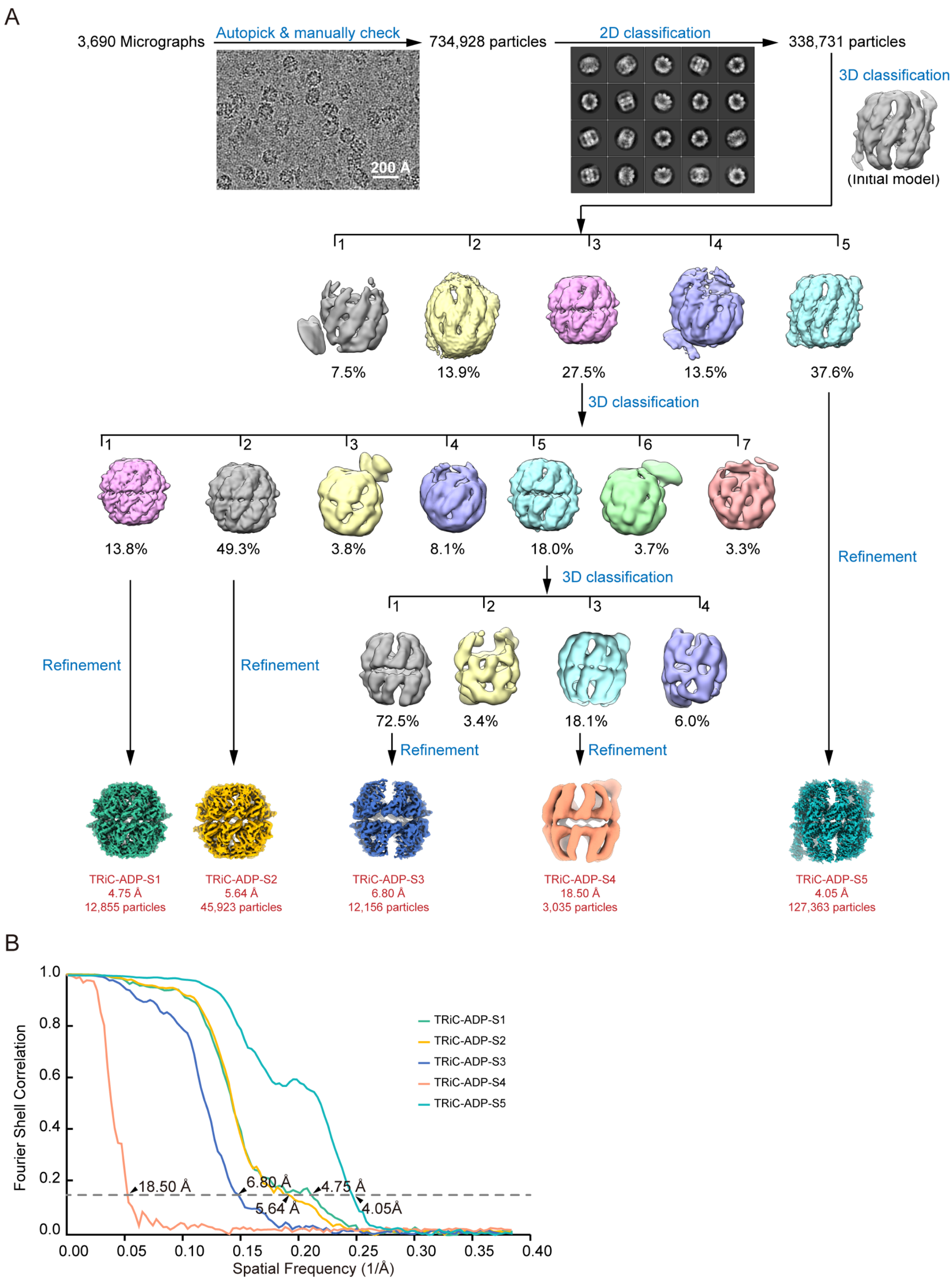

C

D

Fig. S1 Workflow for the processing of cryo-EM data of TRiC and resolution evaluations of map. (A) 3D classification and refinement procedures of TRiC 1 mM ADP. (B) Resolution estimations of the TRiC maps according to the gold-standard FSC criterion of 0.143. (C-D) The Resmap-derived local resolution estimation of TRiC-ADP-S1 and TRiC-ADP-S5 are also shown.

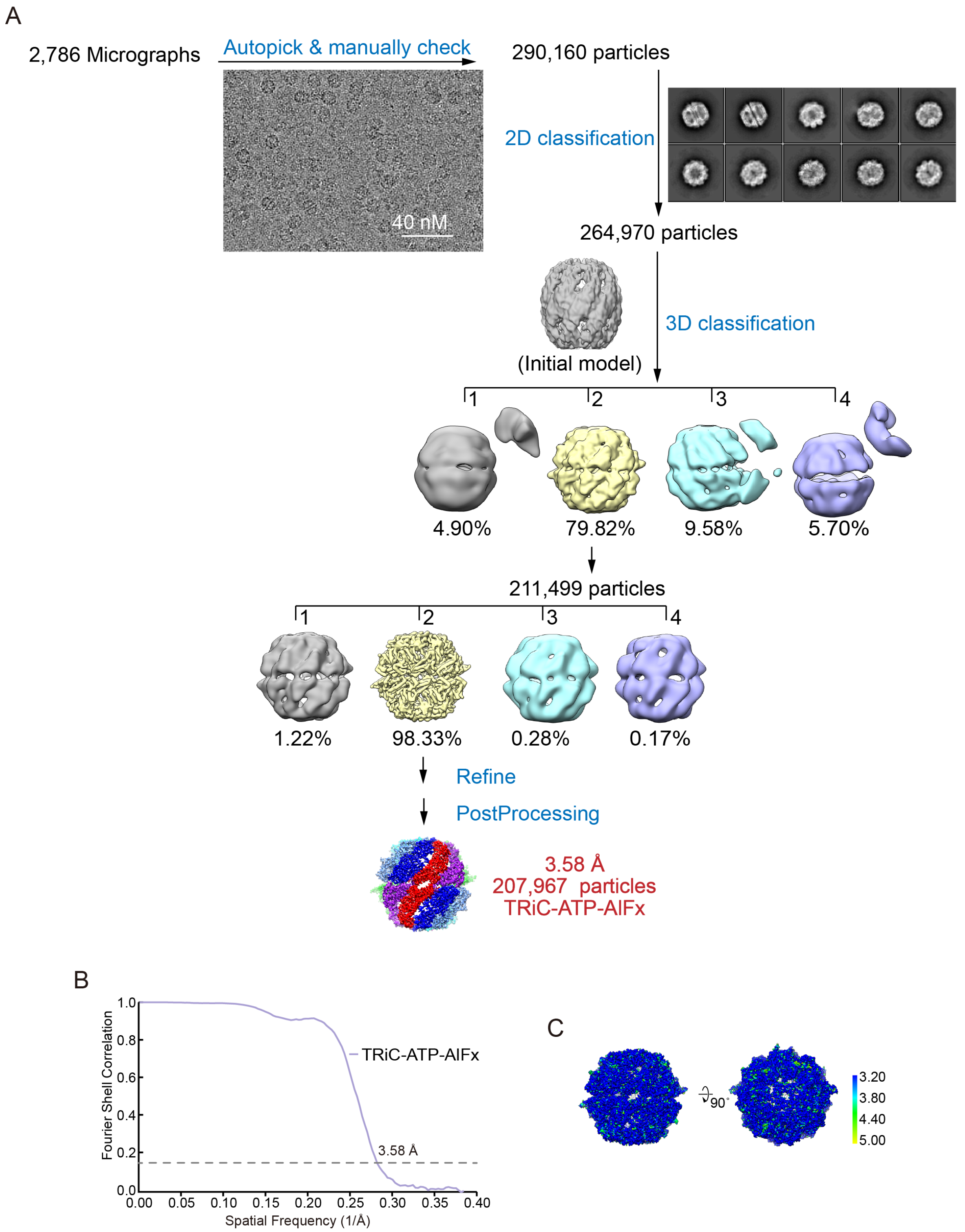

Fig. S2 Work flow for the processing of cryo-EM data of TRiC and resolution evaluations of maps. (A) 3D classification and refinement procedures of TRiC in the presence of 1 mM ATP-AlFx. (B) Resolution estimations of the TRiC maps according to the gold-standard FSC criterion of 0.143. (C) The Resmap-derived local resolution estimation of TRiC-ATP-AlFx.

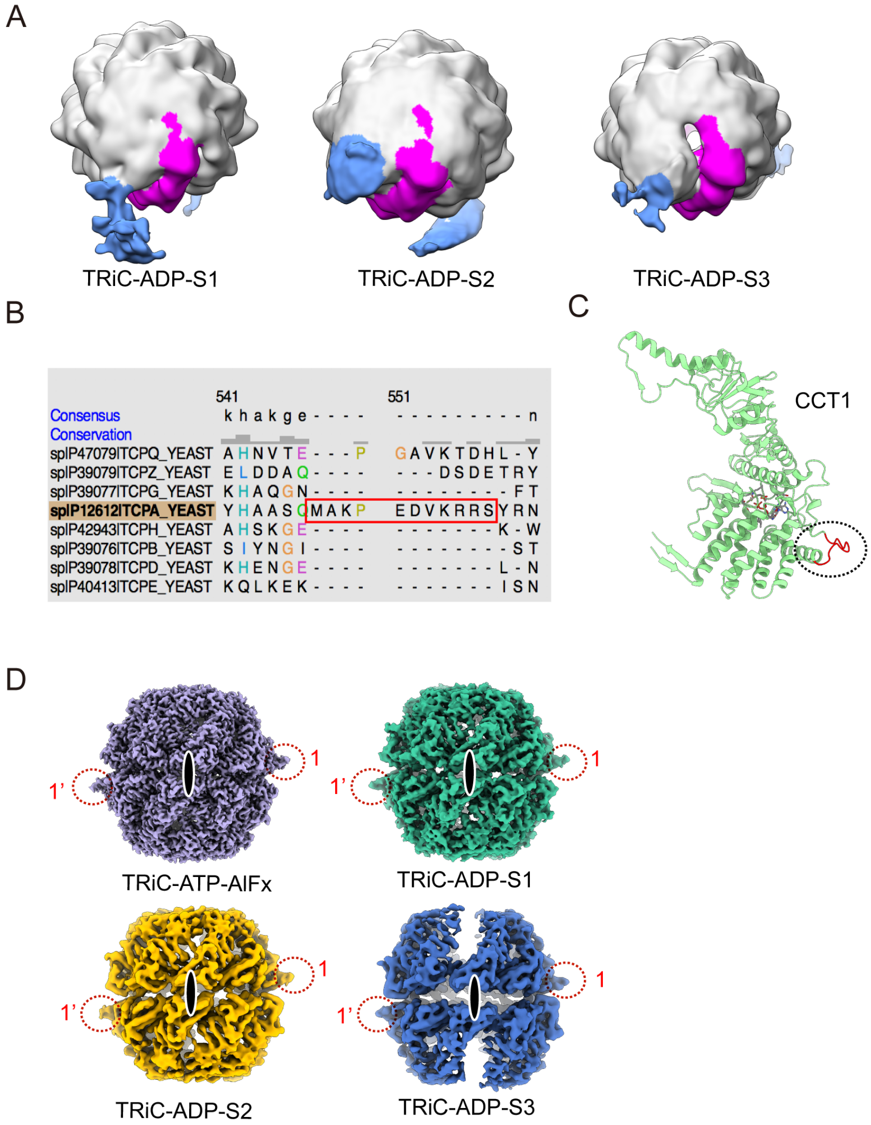

Fig. S3 Determination of the subunit arrangement of TRiC-ADP. (A) The affinity label CBP on the cis and trans rings of CCT3 subunits can be interpreted through the TRiC-ADP-S1, TRiC-ADP-S2, and TRiC-ADP-S3 maps in the early stages of data processing, revealing the relative position of CCT3 subunits in these maps. (B-C) Sequence alignment of eight subunits of yeast TRiC, revealing a unique insertion (marked by red frames) in the E domain of CCT1 (C), with the location of this CCT1 insertion (in red, highlighted by a dotted black ellipsoid) shown in the CCT1 structure (C). (D) The outmost CCT1 insertion in the E domain (indicated by the dashed red circle) from both rings is visible in the TRiC-ATP-AlFx, and TRiC-ADP-S1/S2/S3 maps. The two-fold symmetry between the two rings is indicated by a black ellipsoid.

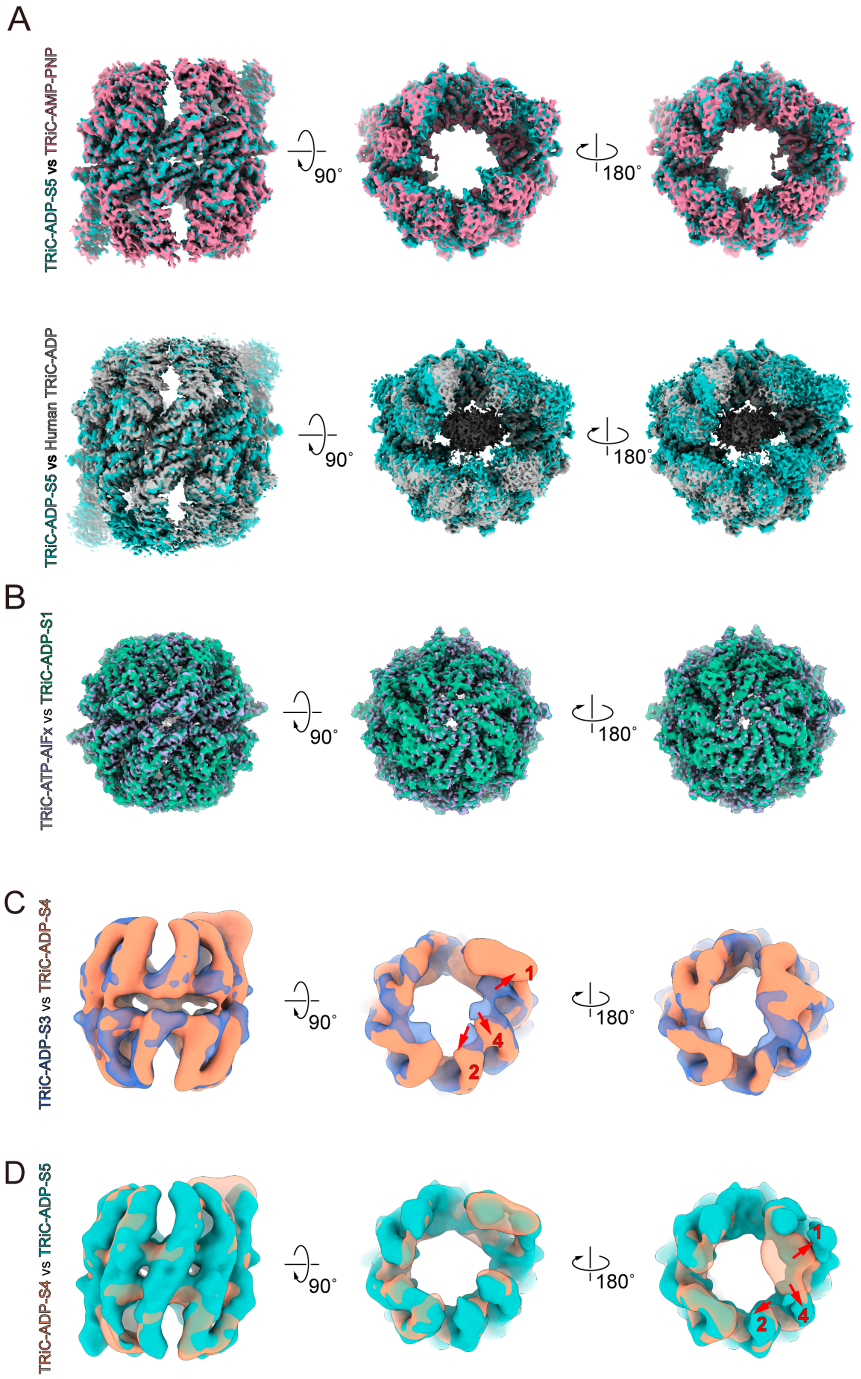

Fig. S4 The conformational comparation between TRiC maps. (A) Upper panel: the structures of TRiC-ADP-S5 and TRiC-AMP-PNP (EMD-9541) are highly similar. Lower panel: the structures of TRiC-ADP-S5 and human TRiC-ADP (EMD-32993) are also very similar. (B) The structures of TRiC-ATP-AlFx and TRiC-ADP-S1 are nearly identical. (C) The trans-ring of TRiC-ADP-S4 remains in a conformation similar to that of the S3 state. Maps were low-pass filtered to 20 Å. (D)The cis-ring of TRiC-ADP-S4 has opened further, resembling the conformation of the open S5 state. Maps were low-pass filtered to 20 Å.

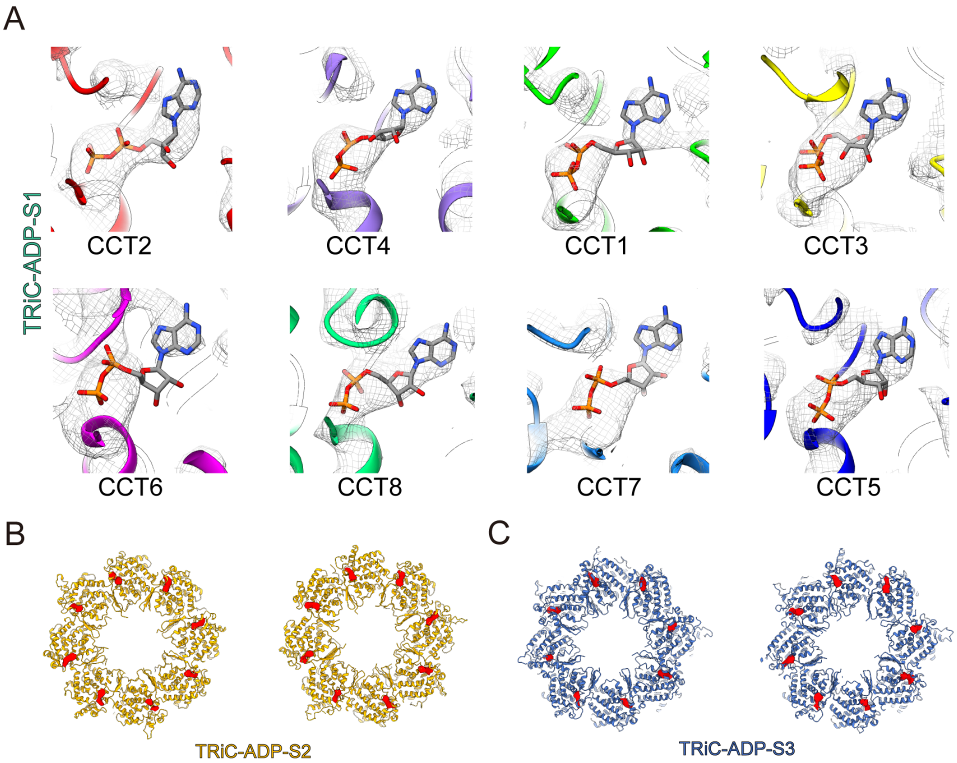

Fig. S5 Nucleotide occupancy statuses of TRiC-ADP structures. (A) A zoomed-in view of the nucleotide pockets in all subunits of the TRiC-ADP-S1 state. (B-C) Nucleotide occupancy statuses in TRiC-ADP-S2 (B) and TRiC-ADP-S5 (C) states. Shown are a slice of the model near the nucleotide pocket region and the nucleotide densities (in red).

Table S1. Statistics of cryo-EM data collection, processing, and model validation.

Conformation	TRiCA-DP-S1	TRiCA-DP-S2	TRiCA-DP-S3	TRiCA-DP-S4	TRiCA-DP-S5	TRiC-ATP-AlFx
Defocus range (μm)	-0.8 to -3.0					-1.2 to -2.0
Final pixel size (Å/pix)	1.318					
Electron dose (e⁻/Å²)	38					
Particles processed	734,928					290,160
Particles refined	12,855	45,923	12,156	3,035	127,363	207,967
Final resolution (Å)	4.75	5.64	6.80	18.50	4.05	3.58
MolProbity score	1.67	1.77	1.63	/	1.30	1.66
Clashscore	7.73	6.91	5.85	/	2.22	7.64
Poor rotamers (%)	0.01	0.01	0.00	/	0.00	0.00
Ramachandran Favored (%)	96.35	95.25	95.51	/	95.71	96.36
Ramachandran Allowed (%)	3.48	4.60	4.34	/	4.07	3.47
Ramachandran Disallowed (%)	0.17	0.15	0.15	/	0.22	0.17
